# A 7-year retrospective study: comparison of the efficacy between rotational osteotomy at the base of femoral neck and vascularized iliac bone flap transfer in the treatment of osteonecrosis of the femoral head

**DOI:** 10.3389/fsurg.2025.1639080

**Published:** 2025-09-25

**Authors:** Tianwei Xia, Jiahao Sun, Lu Wang, Shanbin Zheng, Yukun Li, Muzhe Li, Tianchen Zhang, Tianjie Hu, Jirong Shen, Yong Ma

**Affiliations:** 1 Jiangsu Province Hospital of Chinese Medicine, Affiliated Hospital of Nanjing University of Chinese Medicine, Nanjing, China; 2 Nanjing University of Chinese Medicine, Nanjing, China

**Keywords:** osteonecrosis of the femoral head, hip preservation, rotational osteotomy, vascularized iliac bone flap transfer, DCE-MRI

## Abstract

**Objective:**

To compare the clinical efficacy of rotational osteotomy at the base of femoral neck and iliac bone flap transfer with ascending branch of lateral femoral circumflex artery(vascularized iliac bone flap transfer) in the treatment of ARCO III stage, CJFH-L type osteonecrosis of femoral head.

**Methods:**

We compared 25 cases (Group A), in which 25 hips underwent rotational osteotomy at the base of femoral neck and 25 cases (Group B), in which 25 hips underwent vascularized iliac bone flap transfer in our hospital from September 2017 to March 2024.The operative time and hospitalization time were compared, the collapse and repair of femoral head and the healing of osteotomy were evaluated**,** and the reasons of failure were analyzed.

**Results:**

All operations were conducted successfully in both groups. The follow-up time was 36.2 ± 21.62 months. The operation time in Group A was (215.20 ± 56.80) minutes, which was longer than that in Group B (183.96 ± 32.14) minutes (*P* = 0.022). There was significant difference in the hospitalization time between the two groups. There was no significant difference in Harris scores between Group A and group B before operation (*P* = 0.831). However, there was significant difference in Harris scores after 3 months, 6 months and at the last follow-up (all *P* < 0.05) in Group A and Group B. The excellent and good rate of Group A was 36% (9 hips), the good rate of Group B was 44% (11 hips). During the longest 7-year follow-up period, 6 hips in Group A and 4 hips in Group B underwent arthroplasty. In addition, one hip in group A underwent total hip exclusion because of infection. DCE-MRI showed that abnormal hyperperfusion of the repaired area was alleviated, and the blood supply of the necrotic area was restored.

**Conclusion:**

Both rotational osteotomy of the base of the femoral neck under surgical dislocation and vascularized iliac bone flap transfer can obtain satisfactory short-term results in the treatment of CJFH-L type, ARCO III stage osteonecrosis of the femoral head. Rotational osteotomy at the base of femoral neck under surgical dislocation can be chosen to delay the collapse process for CJFH-L2 type, ARCOⅢB stage ONFH. Vascularized iliac bone flap transfer has the advantage of short operation time. It is easier and suitable for CJFH-L1 type, ARCOⅢA stage ONFH.

## 1 Introduction

Osteonecrosis of the femoral head (ONFH) is a kind of hip disease with a high disability rate. It often progresses gradually, causing persistent hip pain and restricted movement in patients, and if not treated in time, it may lead to severe hip dysfunction and even disability. Considering the limited life of total hip prosthesis, hip-preserving treatment for young and middle-aged patients has great significance (1). The condition of collapse and repair in weight-bearing area is imbalanced in ARCO III stage (2). At this time, self-repair in femoral head is relatively active, which is suitable for hip-preserving operation. However, there are many hip preservation methods for ARCOIII stage ONFH, such as core decompression, bone grafting, iliac bone flap transfer, rotational osteotomy and so on. In recent years, two of these techniques have received increasing clinical attention, though their direct comparison remains lacking. The first is femoral neck base rotational osteotomy, which evolved from transtrochanteric rotational osteotomy. The rotational osteotomy of the base of the femoral neck was developed from transtrochanteric rotational osteotomy, which was improved by Zhang Hong according to the technique of surgical dislocation of the hip and the release of soft tissue flap (3–5). Internationally, early transtrochanteric rotational osteotomy showed promise in load redistribution but faced issues with complications and complexity (3). A good weight-bearing area of the femoral head can be obtained by osteotomy on the premise of protecting the blood supply of the femoral head and rotating the anterolateral necrotic area to the unbearing side (3–5). However, rotational osteotomy is difficult, massive blood loss, and needs a long recovery time—factors restricting its use in low-volume centers.

The second technique is vascularized iliac bone flap transfer with the ascending branch of the lateral circumflex femoral artery. Iliac bone flap transfer with the ascending branch of the lateral circumflex femoral artery(vascularized iliac bone flap transfer) aims to reconstruct the internal structure of the femoral head with the help of fresh blood supply and strong mechanical support by removing necrotic bone and implanting a graft with a nutrient artery (6). Internationally, early studies have shown that this technique has a certain effect in improving the blood supply of the femoral head, but there are differences in the reported surgical effects, which may be related to the patient's condition, surgical operation and postoperative rehabilitation. Domestically, many clinical institutions have carried out this surgery, and some studies have found that it can effectively reduce the progression of femoral head necrosis in some patients, but the effect in patients with severe collapse is not as good as expected (6). However, the applicability of this procedure in patients with femoral head collapse remains controversial, and studies have found that there is bone resorption after the procedure, and that the subchondral bone does not integrate effectively with the implanted bone, resulting in failure of hip preservation (6). In recent years, two kinds of hip-sparing surgery(surgeries) for ONFH have been reported, but there is no comparison between them. Moreover, there is a lack of analysis of patients with failed hip preservation. In this study, two kinds of hip-preserving surgery were used to treat ONFH in ARCO III stage, the surgical effects and failed cases were evaluated from hip function(Harris score),morpholgy of feomral head via x-ray, CT, MRI, blood supply of feomral head via DCE-MRI, in order to provide a reference for clinical treatment selection.

## 2 Data and methods

### 2.1 Ethical approval

This retrospective study was approved by the Medical Ethics Committee of the Affiliated Hospital of Nanjing University of Chinese Medicine(2023NL-058-02)and was registered with the China Clinical Trial Registry (ChiCTR1900024981, registration date: 2019/08/06). After all patients were informed of the surgical procedure, their written informed consent was obtained. All procedures are carried out in accordance with the “Helsinki Declaration” of WHO.

### 2.2 Patient selection criteria

Inclusion criteria: (1) patients aged from 18 to 50 years old; (2) patients who met the criteria of ARCO IIIA, IIIB (1994 edition) and lateral type in China-Japan Friendship Hospital (CJFH-L) classification; (3) the patients were treated with rotational osteotomy at the base of femoral neck under surgical dislocation or iliac bone flap transfer with ascending branch of lateral circumflex femoral artery;(4) follow-up is more than 6 months.

The exclusion criteria were as follows: (1) the weight-bearing area of the femoral head collapsed seriously (>4 mm), (2) the range of motion of the hip joint was severely restricted, including flexion <90°, abduction <30°, adduction <30°, (3) patients with lower limb deformities., (4) patients with severe liver and kidney diseases, (5) patients who underwent hip-preserving surgery on both sides at the same time.

A total of 50 patients (50 hips) were included in this study. 25(25 hips) underwent rotational osteotomy at the base of the femoral neck under surgical dislocation (Group A), and 25 cases (25 hips) were treated with iliac bone flap transfer with ascending branch of lateral circumflex femoral artery (Group B).

The required sample size was determined *a priori* using the standard formula for two independent samples comparing continuous outcomes:

$$n=\frac{2\times {\left(Z_{1-\alpha /2}+Z_{1-\beta }\right)}^{2}\times \sigma^{2}}{\delta^{2}}$$

Parameters were specified as follows: Minimal clinically important difference (δ): 10 points on the Harris Score, consistent with established thresholds for functional improvement in osteonecrosis trials. Standard deviation (σ): 12 points, derived from pilot data (*n* = 10) and corroborated by published studies.

Type I error rate (α): 0.05 (two-tailed). Statistical power (1-β): 80%.

This calculation yielded a requirement of 23 participants per group. Accounting for an anticipated 10% attrition rate, the adjusted sample size was 25 participants per group (total *N* = 50). The final cohort comprised 25 patients who underwent rotational osteotomy (Group A) and 25 patients receiving iliac bone flap transfer (Group B), totaling 50 participants. *post-hoc* power analysis using observed data (pooled SD = 14.6, mean *Δ*Harris Score = 8.2) demonstrated 80% power to detect the predefined 10-point difference at *α* = 0.05.

### 2.3 General information

Group A: 22 male (22 hips), 3 female (3 hips), age 19–45 years (31.54 ± 6.93), BMI 17.5–31.1(24.0 ± 3.7), course of disease 2–20 months (8.52 ± 6.27), left hip 13 cases, right hip 12 cases, ONFH type: idiopathic 12 cases (12 hips), steroid 3 cases (3 hips), alcohol 7 cases (7 hips), traumatic 3 cases (3 hips). ARCO stage: 10 hips in Stage IIIA and 15 hips in stage IIIB. According to CJFH classification, there were 13 hips of L1, 10 hips of L2 and 2 hips of L3.

Group B: 20 Male (20 hips), 5 female (5 hips), age 24.48 years (32.36 ± 6.39), BMI 17.2231.56 (24.84 ± 3.90), course of disease 1–25 months (7.64 ± 7.76), left hip 16 cases, right hip 9 cases, ONFH type: idiopathic 7 cases (7 hips), steroid 8 cases (8 hips), alcohol 8 cases (8 hips), traumatic 2 cases (2 hips). ARCO stage: 9 hips in Stage IIIA, 16 hips in stage IIIB. According to CJFH classification, there were 14 hips in Type L1, 10 hips in Type L2 and 1 hip in Type L3.

### 2.4 Surgical methods

**Figure 1:**
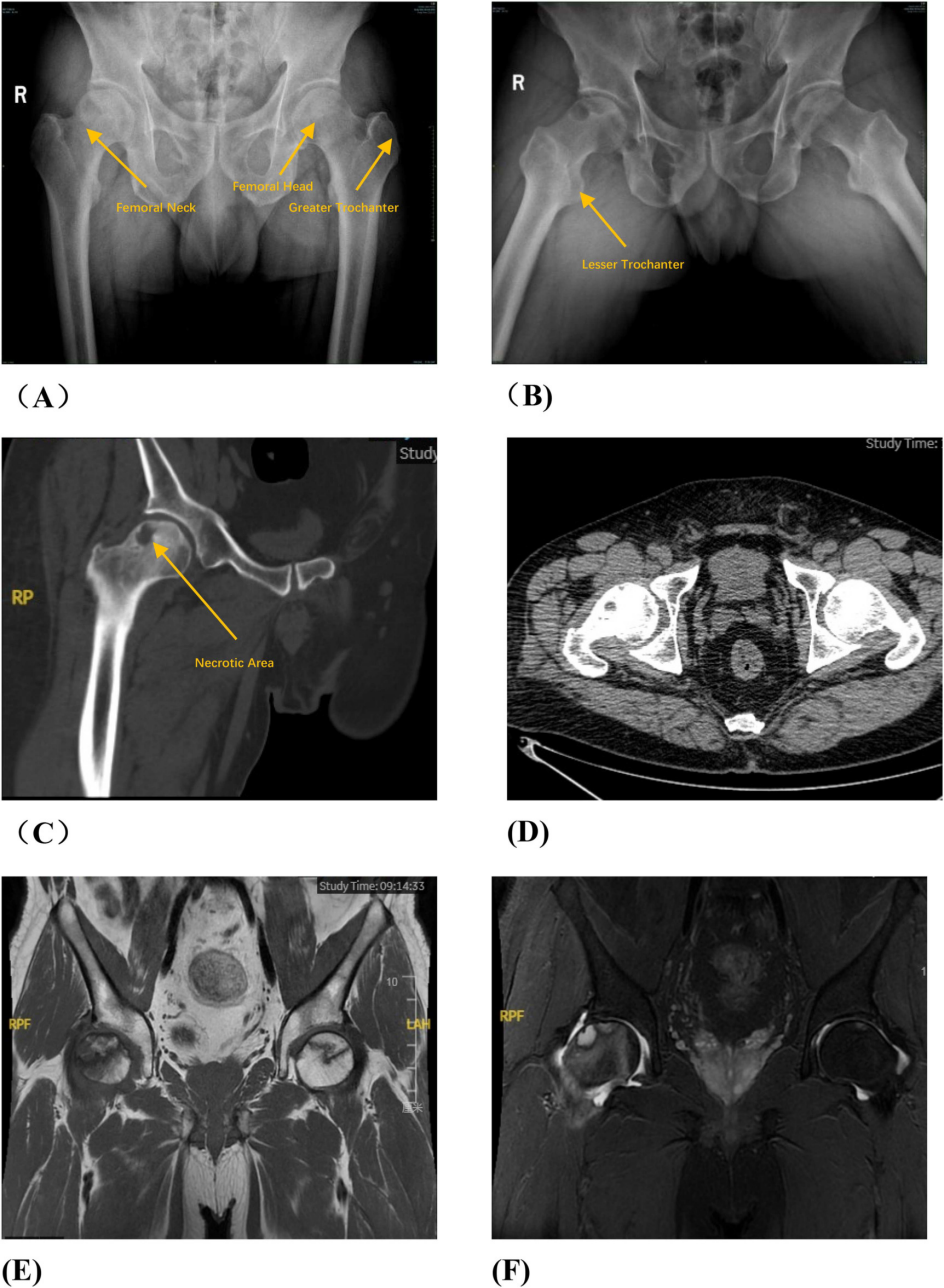
Preoperative planning for rotational osteotomy. **(A)** Preoperative anteroposterior (AP) radiograph. **(B)** Preoperative frog radiograph. **(C,D)** Preoperative 256-row CT. **(E)** Preoperative T1MRI. **(F)** Preoperative T2MRI; **(G)** Three-dimensional modeling after CT and MRI alignment. **(H)** Measurement of necrotic area angle. **(I–K)** Determination of the osteotomy plane to ensure that the necrotic area leaves its lateral side after rotation, with yellow indicating the necrotic area **(I)** distance from the osteotomy line to the apex of the femoral head. **(J)** distance from the upper edge of the osteotomy to the greater trochanter.K: distance from the lower edge of the osteotomy to the lesser trochanter). **(L)** Top view after 130° of simulated right lateral rotation. **(M)** Simulated result of left lateral rotational osteotomy.

**Figure 2:**
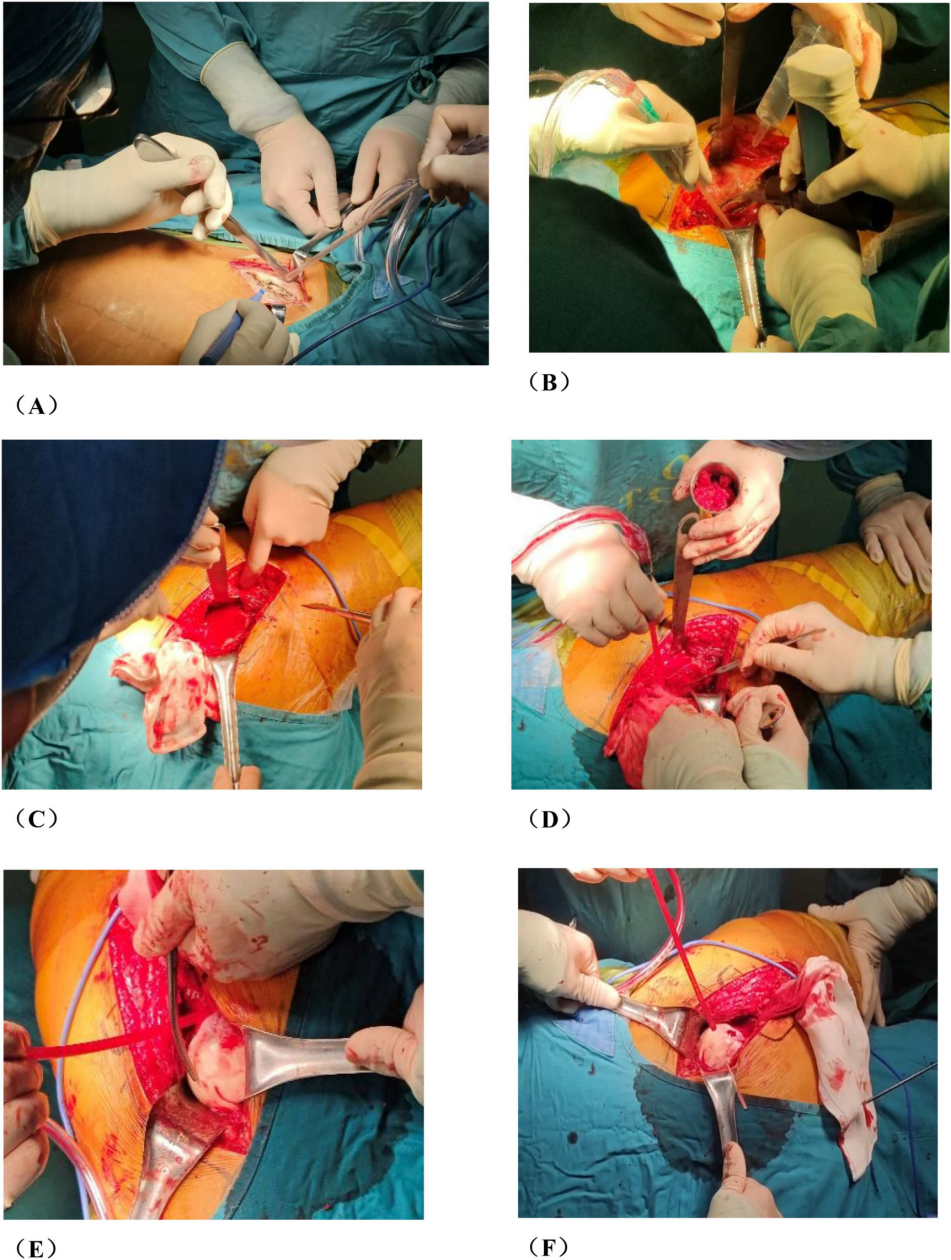
Photos of rotational osteotomy at the base of femoral neck under surgical dislocation during operation. **(A)** Taking the lateral incision of the hip joint to incise. **(B)** Osteotomy of the greater trochanter. **(C)** Soft tissue flap lengthening with a bone cutter. **(D)** Soft tissue flap lengthening with a scalpel. **(E)** Surgical dislocation to reveal the femoral head. **(F)** Drilling of Kirschner's wire to further determine the location of necrotic area by the presence or absence of hemorrhage. **(G)** Osteotomy of the femoral neck at its base. **(H)** Temporary fixation with a Kirschner's wire. **(I)** Fluoroscopic rotation under fluoroscopy to the appropriate angle, preserving a good lateral column. **(J)** Femoral neck system(FNS) was usually used to fix the femoral neck osteotomy.

**Figure 3:**
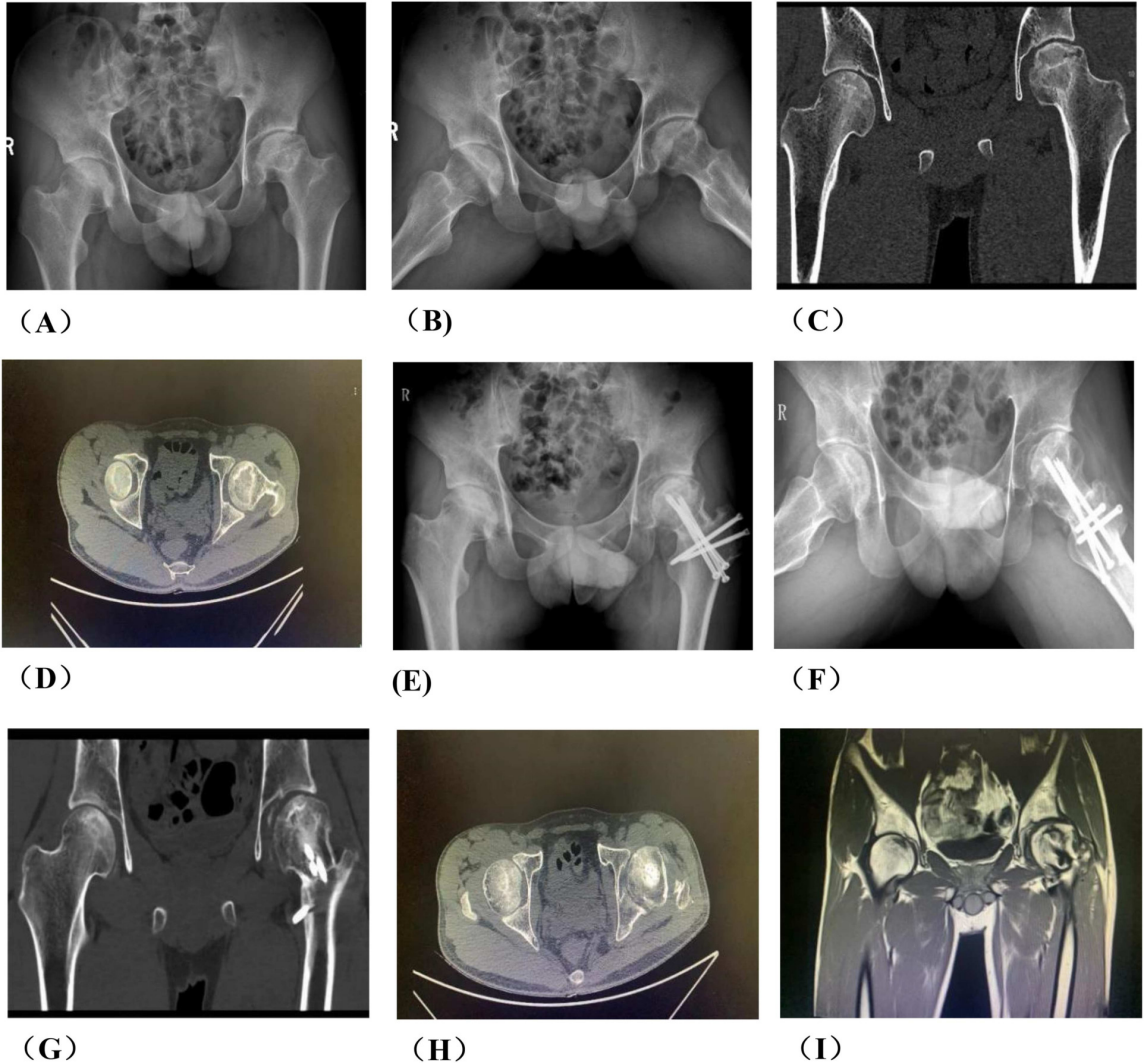
A typical case in group A, patient wang, a 19-year-old male with left osteonecrosis of the femoral head, ARCO stage III B. **(A,B)**. Preoperative x-rays; **(C,D)**. Preoperative CT; **(E,F)**. x-rays at 12 months postoperative; **(G,H)**. CT at 12 months postoperative; **(I)** MRI at 12 months postoperative.

Group A: The patient was placed in a lateral position after general anesthesia. We made the lateral incision of affected hip joint to expose and protect the insertion points of the gluteus medius and rectus femoris muscles. Subsequently, the greater trochanteric osteotomy is performed while removing approximately one-third of its cancellous bone from its posterior aspect. Then, opening along the osteotomy site towards the anterior direction exposes both the femoral neck and joint capsule.We used a retractor to dislocate the femoral head forward, observed the anterolateral collapse of the femoral head. A Kirschner wire was used to drill, aimed to explore the blood supply of the femoral head. After fully exposing the base of the femoral neck, we sawed femoral neck with an electric saw. After temporarily fixing the femoral head with a Kirschner wire, we rotated the femoral head forward or backward. We performed intraoperative fluoroscopy to ensure that the necrotic area was removed to obtain a satisfactory anterolateral weight-bearing area. Three cannulated screws or femoral neck system(FNS) was usually used to fix the femoral neck osteotomy, and fluoroscopy was used to ensure that the internal fixation was firm. Then we reduced the hip and fixed the greater trochanter with two cannulated screws. Finally, we sutured the capsule and closed the incision layer by layer. The preoperative planning is shown in Figure 1, the operative photos in Figure 2, and the typical case in Figure 3.

**Figure:**
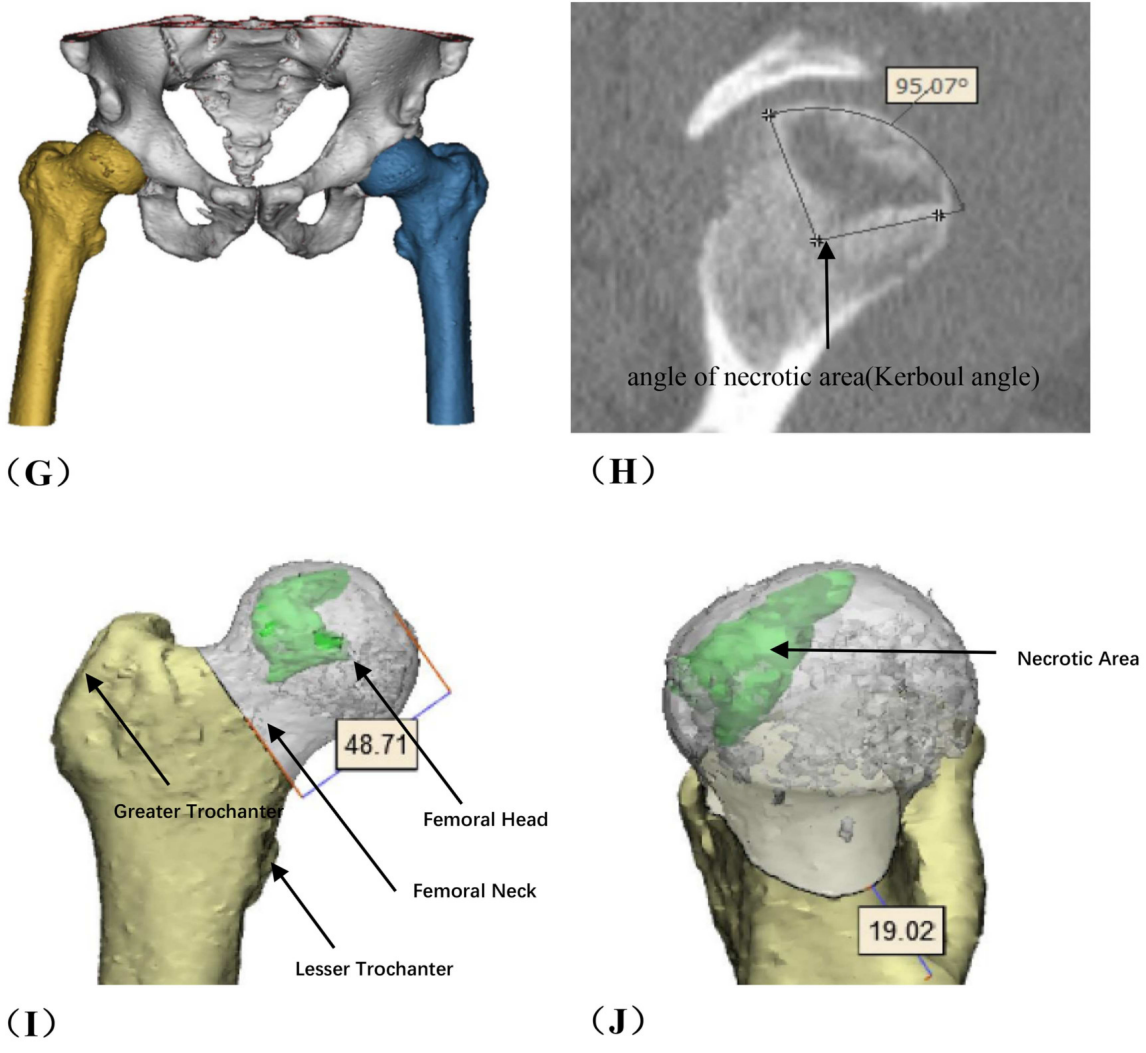


**Figure:**
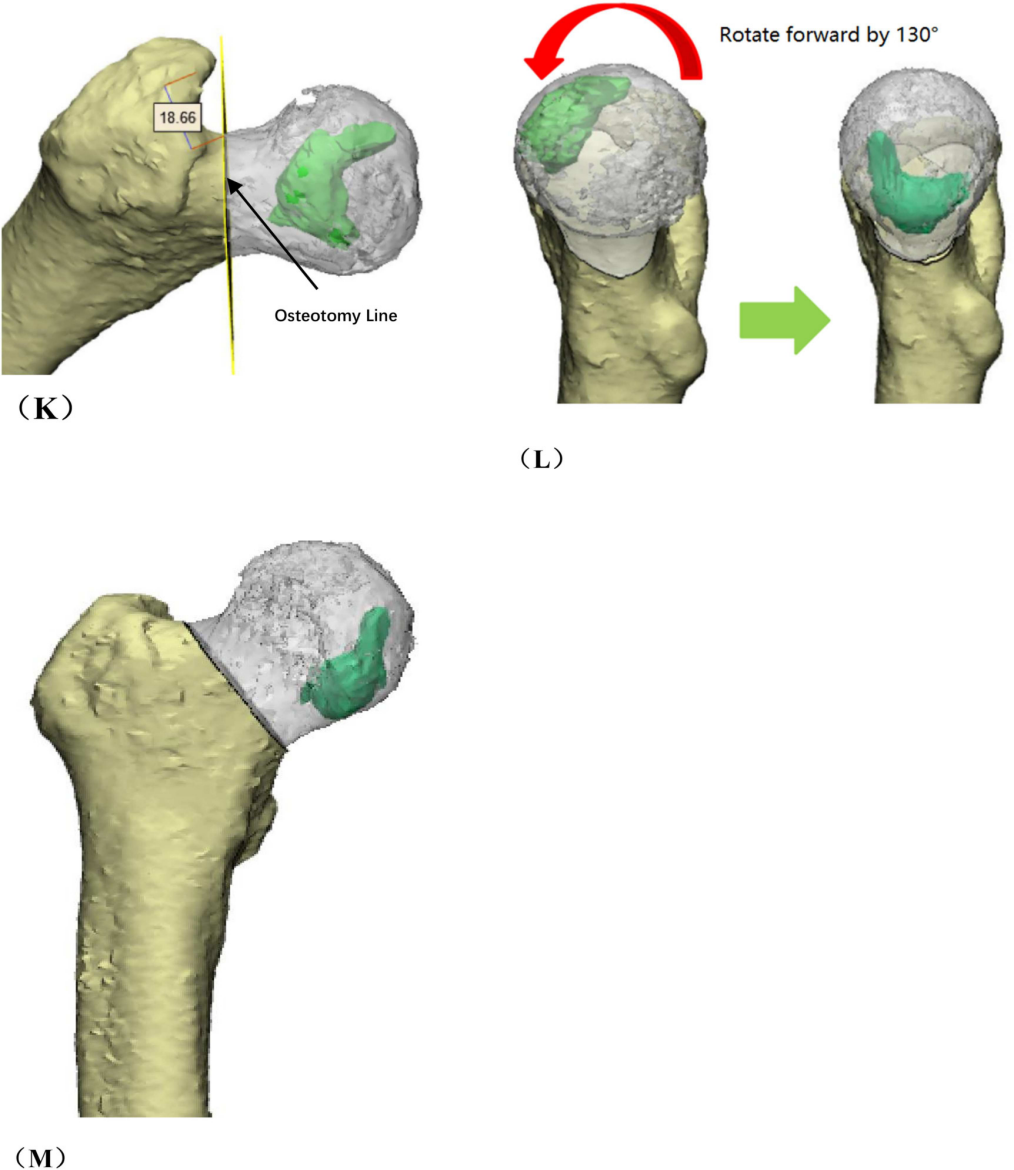


**Figure:**
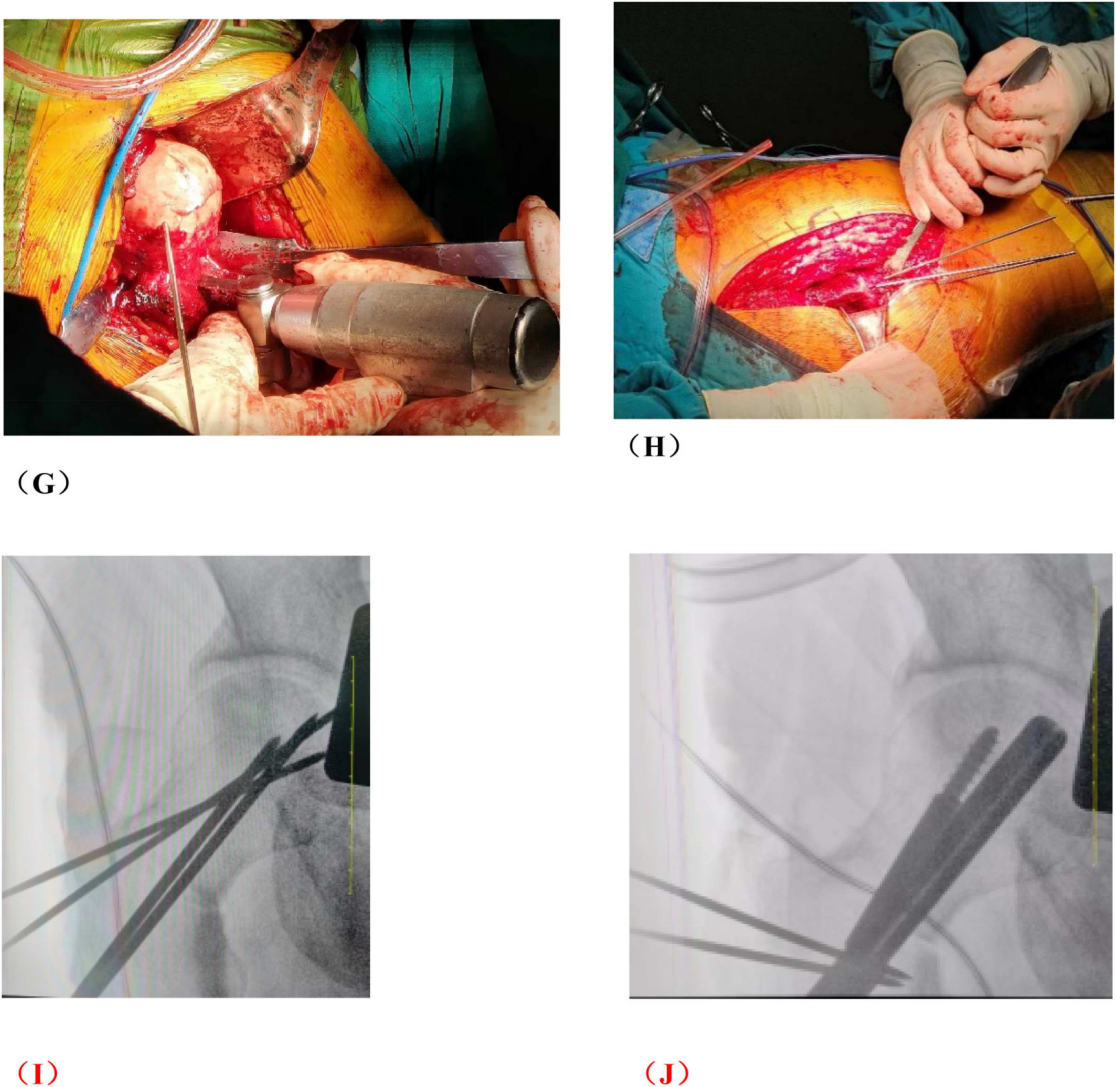


**Figure 4:**
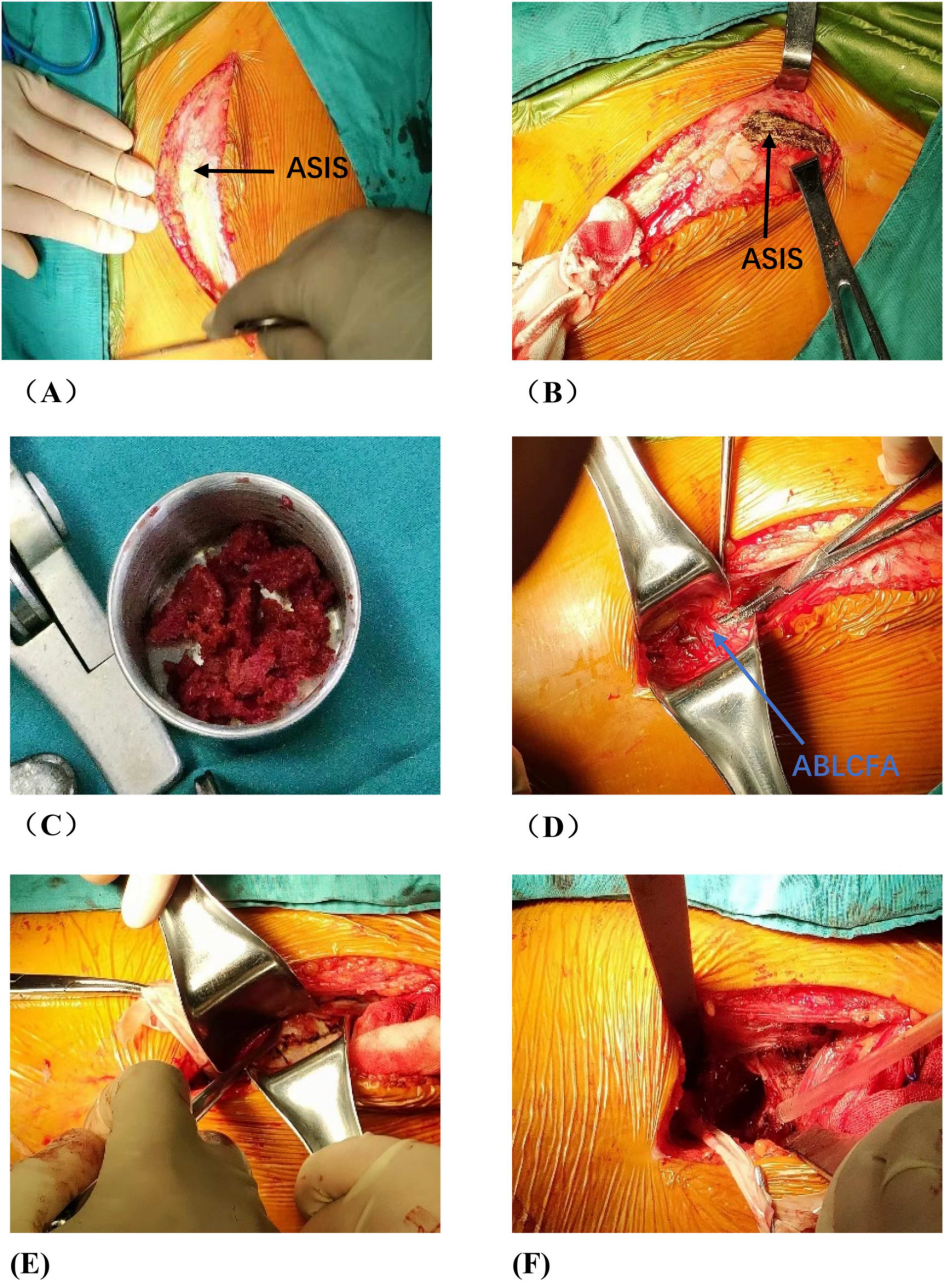
Photos of vascularized iliac bone flap transfer during operation. The patient was traumatic osteonecrosis of the femoral head. Three cannulated screws were fixed before this operation. **(A)** SP incision. **(B)** We take the cortical bone above the anterior superior iliac spine in the external plate of the ilium. **(C)** We excavated the cancellous bone. **(D)** We separated the ascending branch of the lateral circumflex femoral artery. **(E)** We cut the capsule to expose the head and neck. **(F)** We removed the sclerotic bone by high-speed abrasive drill, and removed necrotic bone by scraping with a curet. **(G)** We satisfactorily removed dead bone from necrotic area as seen by fluoroscopic view. We find three cannulated screws were secure, so we did not extract them during operation. **(H)** The necrotic area was filled with cancellous bone and pressed. The iliac bone flap of the tensor fascia latae with the ascending branch of the lateral circumflex femoral artery was detached and transferred to necrotic area. **(I)** The cortical bone of the external plate of the ilium with abundant blood supply was further filled in the necrotic area to support the anterolateral column. **(J)** The titanium cannulated screw was used to fix iliac bone graft and we find it was secure under fluoroscopy. ASIS, anterior superior iliac spine; ABLCFA, ascending branch of lateral circumflex femoral artery; IBF, iliac bone flap.

**Figure 5:**
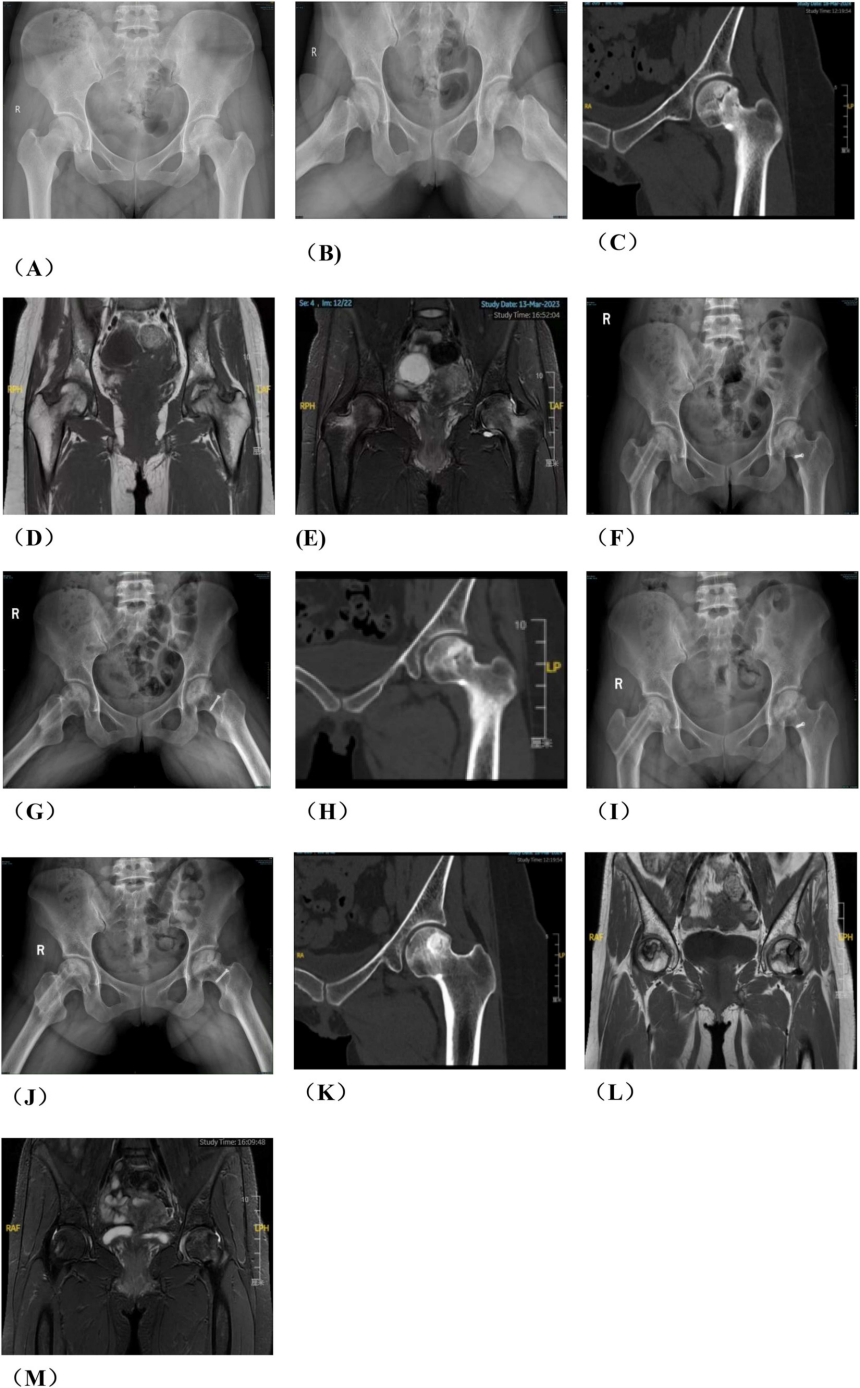
Patient in group B, wang X, female, 29 years old, right hip ONFH, ARCO stage IIIA, **(A)** preoperative x-ray anteroposterior position film, **(B)** preoperative x-ray frog position film; **(C)** Pre-op CT, **(D)** preoperative T1-MRI; **(E)** preoperative T2-MRI; **(F,G)** x-ray 6 months after surgery; **(H)** 6 months CT after operation; **(I,J)** 12 months after operation; **(K)** CT 12 months after operation; **(L)** T1-MRI 12 months after surgery; **(M)** T2-MRI was performed 12 months after operation.

Group B: After general anesthesia, the patient was placed in a supine position and placed on a proper square pad under the operating hip to raise the operating hip joint by 30°. We made a Smith-Petersen incision from the anterior superior iliac spinet to the tensor fasciae latae. Then we used a narrow chisel to cut out the vascularized iliac bone flap (approximately 1.5 cm × 1.5 cm × 2 cm in size). Then we excavated proper cancellous bone. Next, we exposed the spacer between rectus femoris and the tensor fasciae latae, and protected the ascending branch of the lateral femoral circumflex artery. We made an incision in the anterior portion of the hip capsule, exposed the head and neck of the femur, opened the bone window with a narrow chisel, debrided the sclerotic bone with a high-speed drill, removed the necrotic bone with a curet, and ensured that the necrotic bone was cleared as much as possible via fluoroscopy. Next, we fully compressed the graft, implanted the iliac bone graft to support the necrotic area and restore the original structure of the collapsed femoral head. We chose a cannulated screw to fix the iliac bone flap. Close the incision layer by layer. The intraoperative photos are shown in Figure 4, and the typical case are shown in Figure 5.

**Figure:**
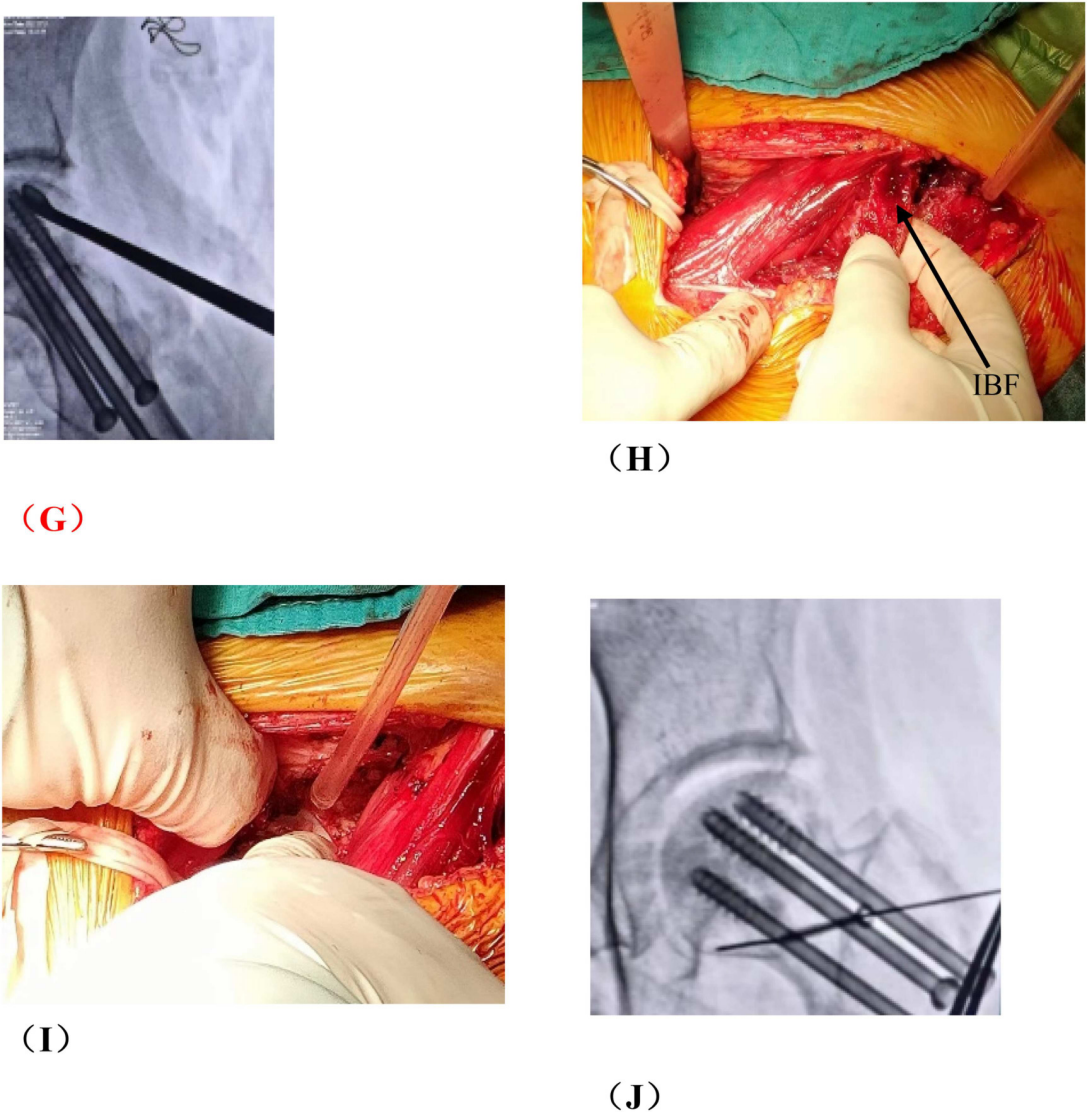


### 2.5 Postoperative treatment

Postoperative routine anti-infection, anti-coagulation, and anti-swelling treatments were given. We guided the patients to do ankle back extension and plantar flexion movement. All patients were bedridden for 3 months. According to the imaging findings, we evaluated the healing of the osteotomy site in Group A and the healing of the bone graft in Group B. If the hip is healing satisfactorily, bilateral axillary crutches were used to keep the affected limbs from weight bearing after 3 months. When approved by the doctor's assessment, the patient used a crutch to partially load the affected limb and could use the affected limb to bear the full weight.

### 2.6 Observation and follow-up

The operation time, length of stay in hospital and postoperative complications were recorded. The effectiveness of the treatment was evaluated by Harris score and imaging examination (including bilateral anteroposterior and frog position film of x-ray, bilateral hip CT and bilateral hip DCE-MRI (7–9). The observation time points included preoperative, 3 months postoperatively and 6 months postoperatively. Harris score: ≥90 indicates excellent function, 80–90 indicates good function, 70–79 indicates fair function, and less than 70 indicates poor function. The safety of the treatment was evaluated by blood routine examination, C-reactive protein (CRP), liver and kidney function, and imaging examination. The observation time points included preoperative, 3 months postoperatively and 6 months postoperatively.

### 2.7 Statistical analysis

SPSS22.0 software was used to analyze the data. The mean ± standard deviation $\left(\bar{x}\pm s\right)$ was used for the measurement data. The independent sample *t* test was used for comparison of the two groups, the paired *t* test was used for the comparison within groups, and the *Χ*^2^ test was used for the counting data, rank sum test was used for rank data. *P* < 0.05 was statistically significant. The survival curves of the two groups were plotted using the Kaplan–Meier survival analysis method.

## 3 Results

### 3.1 Clinical results

#### 3.1.1 Comparison of perioperative data between the two groups

There were no significant differences in sex (*P* = 0.324), age (*P* = 0.880), duration (*P* = 0.557), ONFH type and stage between the two groups (*P* > 0.05) (Table 1). All operations in both groups were successfully conducted, except one in which a patient in Group B reported numbness of the lateral skin of the affected limb. The operation time of Group A was (215.20 ± 56.80) min, which was longer than that of Group B (183.96 ± 32.14) minutes (*P* = 0.022), and length of stay of Group B was longer than that of group A (*P* = 0.036) (Table 2).

**Table 1:** Comparison of baseline data between groups.

Item	Group A	Group B	Statistic	*p*-value
Sex(male/female)	22/3	20/5	OR = 0.55 (0.11–2.76)	0.707
Age(years)	32.04 ± 6.93	32.36 ± 6.39	t = −0.44, df = 48	0.866
BMI(kg/m^2^)	24.0 ± 3.7	24.84 ± 3.90	t = −0.78, df = 48	0.442
Course(months)	6.04 ± 7.95	7.64 ± 7.76	t = 0.44, df = 46	0.475
Side of operation(right/left)	12/13	9/16	*χ*² = 0.74, df = 1	0.390
Types of osteonecrosis(idiopathic, steroid, alcohol, traumatic)	12/3/7/3	7/8/8/2		0.278
ARCO stage(ⅢA/ⅢB)	10/15	9/16		0.999
CJFH type(L1/L2/L3)	13/10/2	14/10/1		0.999

**Table 2:** Comparison of perioperative data between the two groups.

Item	Group A	Group B	Statistic	*p*-value
Operation Time (minutes)	215.20 ± 56.80	183.96 ± 32.14	t = 2.42	0.022
Length of stay (days)	14.04 ± 3.56	18.24 ± 7.38	t = −2.18	0.036

#### 3.1.2 Comparison of clinical follow-up data between the two groups

**Figure 6:**
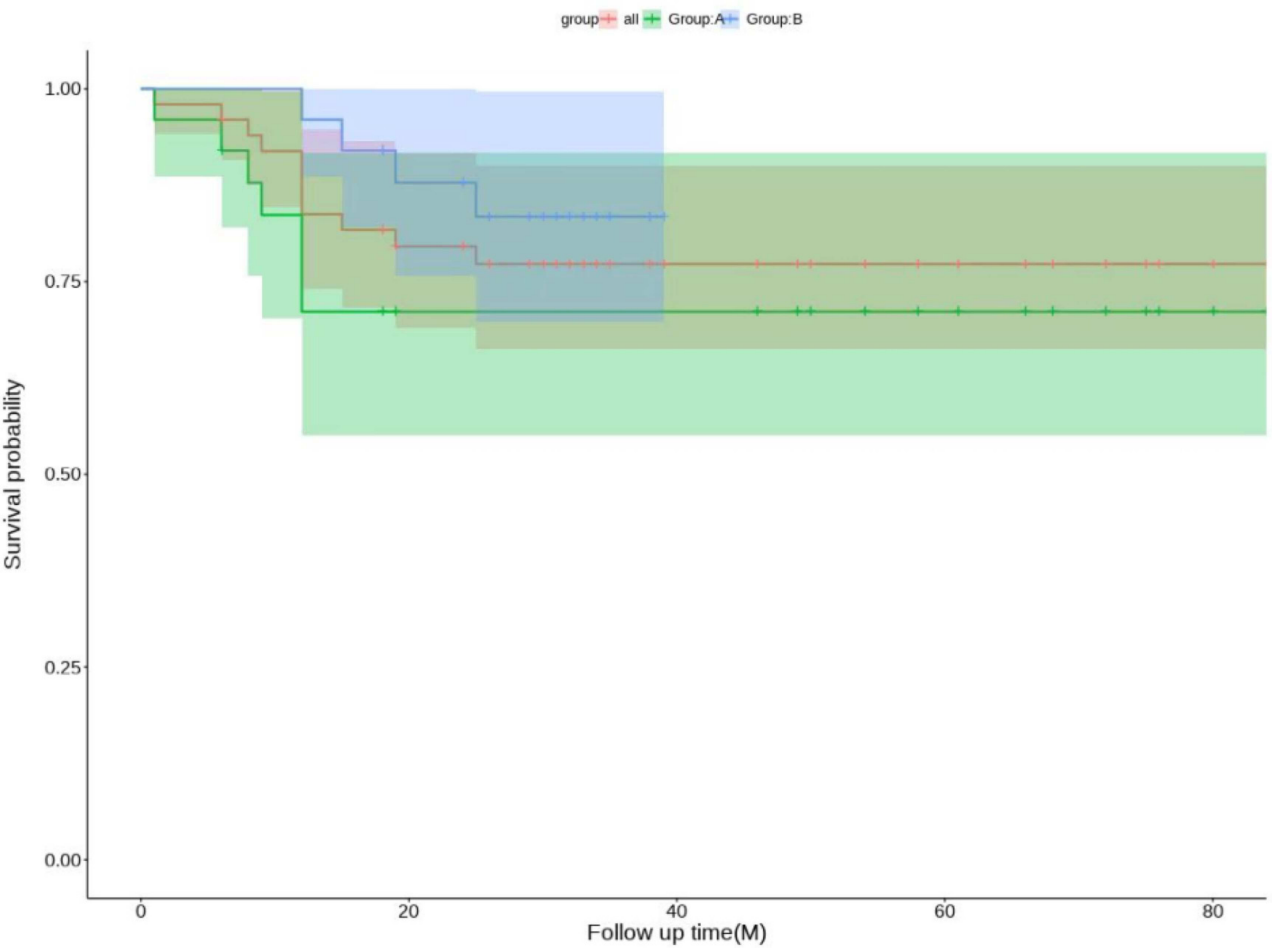
Survival curves for both groups at long-term follow-up.

The patients in both groups were followed up at 3 and 6 months postoperatively. If the hip was preserved successfully, the follow-up was continued. If the hip was not preserved, the follow-up was ended. The follow-up time was 36.20 ± 21.62 months. There was no significant difference in Harris scores between Group A and group B (*P* = 0.831). Compared with preoperative scores, there were statistically significant differences in the scores of patients in Groups A and B at 3 months postoperatively, 6 months postoperatively, and at the last follow-up(*P* < 0.05). Patients in Group B had better Harris scores than those in Group A at 3 months, 6 months and at the last follow-up (Table 3). One patient had internal fixation looseness and underwent total hip arthroplasty, 6 patients had loss of cervical shaft angle and coxa vara, and 5 patients had undergone total hip arthroplasty in Group A. At the 6-month follow-up, the osteotomies of 3 patients were not healed well, and local immobilization was performed. One patient was injected Teriparatide subcutaneously, and all osteotomies healed at the last follow-up. In Group B, 2 patients had fat liquefaction and wound healing after debridement, 1 patient had numbness of the lateral skin of the affected limb, and the other patients had no complications. At the last follow-up, there were 6 total hips in Group A and 4 total hip arthroplasties in Group B. In addition, one hip in Group A underwent total hip exclusion because of hip infection. In summary: Group A got 4 excellent hips, 5 good hips, 9 fair hips, 7 poor hips, excellent and good rate 36%; Group B got 6 excellent hips, 5 good hips, 9 fair hips, 5 poor hips, excellent and good rate 44%. The specific survival curve of the two groups with long-term follow-up is shown in Figure 6.

**Table 3:** Comparison of harris clinical follow-up scores between the two groups.

Time point	Group A	Group B	Statistic	*p*-value
Pre-operation	55.00 ± 6.37	58.33 ± 5.67	t = 1.33	0.190
3 months of Follow-up^a^	65.88 ± 6.13	69.68 ± 6.21	t = −2.06	0.045
6 months of follow-up^a^	71.25 ± 7.80	80.87 ± 9.26	t = −3.21	0.012
Last follow-up^a^	74.33 ± 12.02	84.18 ± 11.29	t = −2.30	0.025

a Harris score of 3 months, 6 months and the last follow-up in Group A and group B were elevated compared with pre-operation.

### 3.2 Comparison of typical patients from two groups one year after operation

#### 3.2.1 DCE-MRI of typical patients in two groups

**Figure 7:**
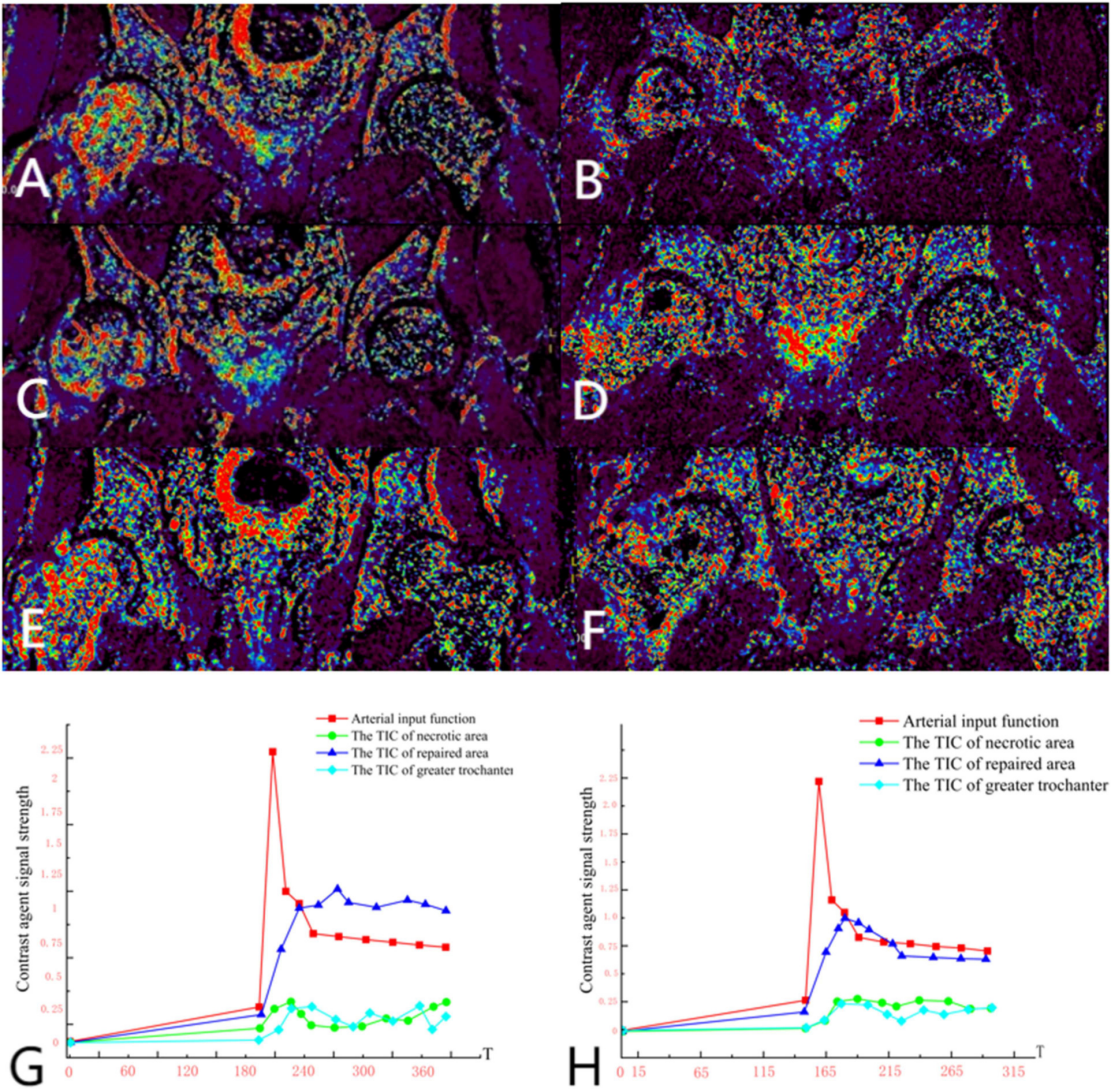
**(A,C,E)** Preoperative DCE-MRI Ktrans pseudocolor maps; **(B,D,F)** postoperative. **(G)** Arterial input function and time-intensity curves (TIC) for necrotic area, repaired area, and greater trochanter preoperatively. **(H)** Arterial input function and time-intensity curves (TIC) for necrotic area, repaired area, and greater trochanter postoperatively. The perfusion in the anterior and posterior necrotic area increased after operation, while the abnormal hyperperfusion in repaired area decreased.

Group A: Ji X, male, 34 years old, osteonecrosis of the left femoral head. The patient underwent left rotational osteotomy in March 2024 (fixed with hollow screws). The patient underwent a follow-up DCE-MRI to assess blood supply six months after surgery (Figure 7).

**Figure 8:**
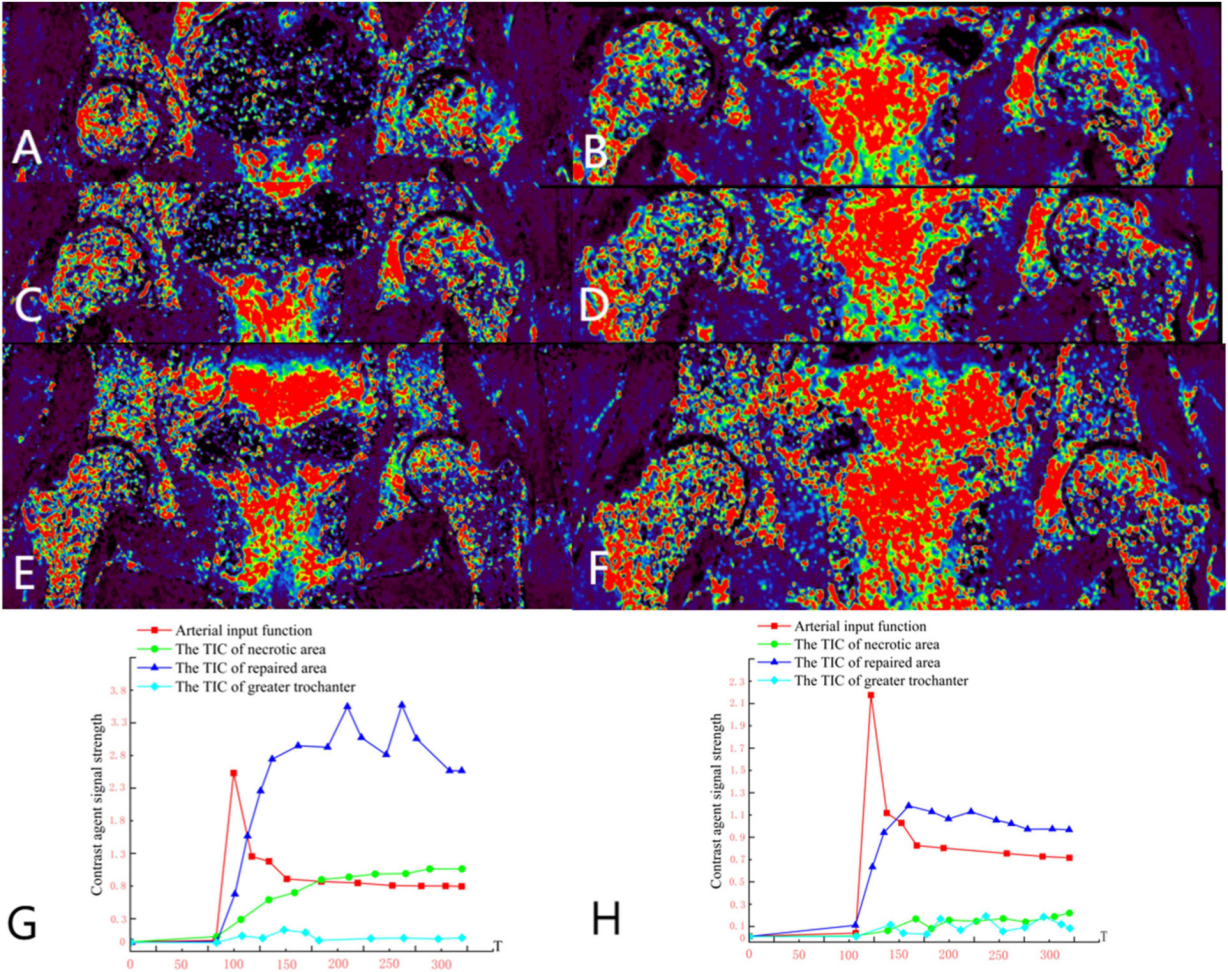
**(A,C,E)** Preoperative DCE-MRI Ktrans pseudocolor maps; **(B,D,F)** postoperative; **(G)** preoperative TIC curve; **(H)** postoperative TIC curve. Preoperative DCE-MRI indicates poor blood perfusion in the right femoral head, with internal instability and a high risk of collapse. The postoperative DCE-MRI shows that compared to the preoperative images, not only the bone marrow edema in the femoral head (sclerotic area) diminished, but also necrotic area shown signs of revascularization.

Group B, Wang X, female, 33 years old, bilateral osteonecrosis of the femoral head. She underwent right PVIBGT (with titanium screw fixed) in March, 2023. We use DCE-MRI to evaluate blood supply of femoral head after 6 months. (Figure 8)

#### 3.2.2 Analysis of patients with failed hip preservation

**Figure 9:**
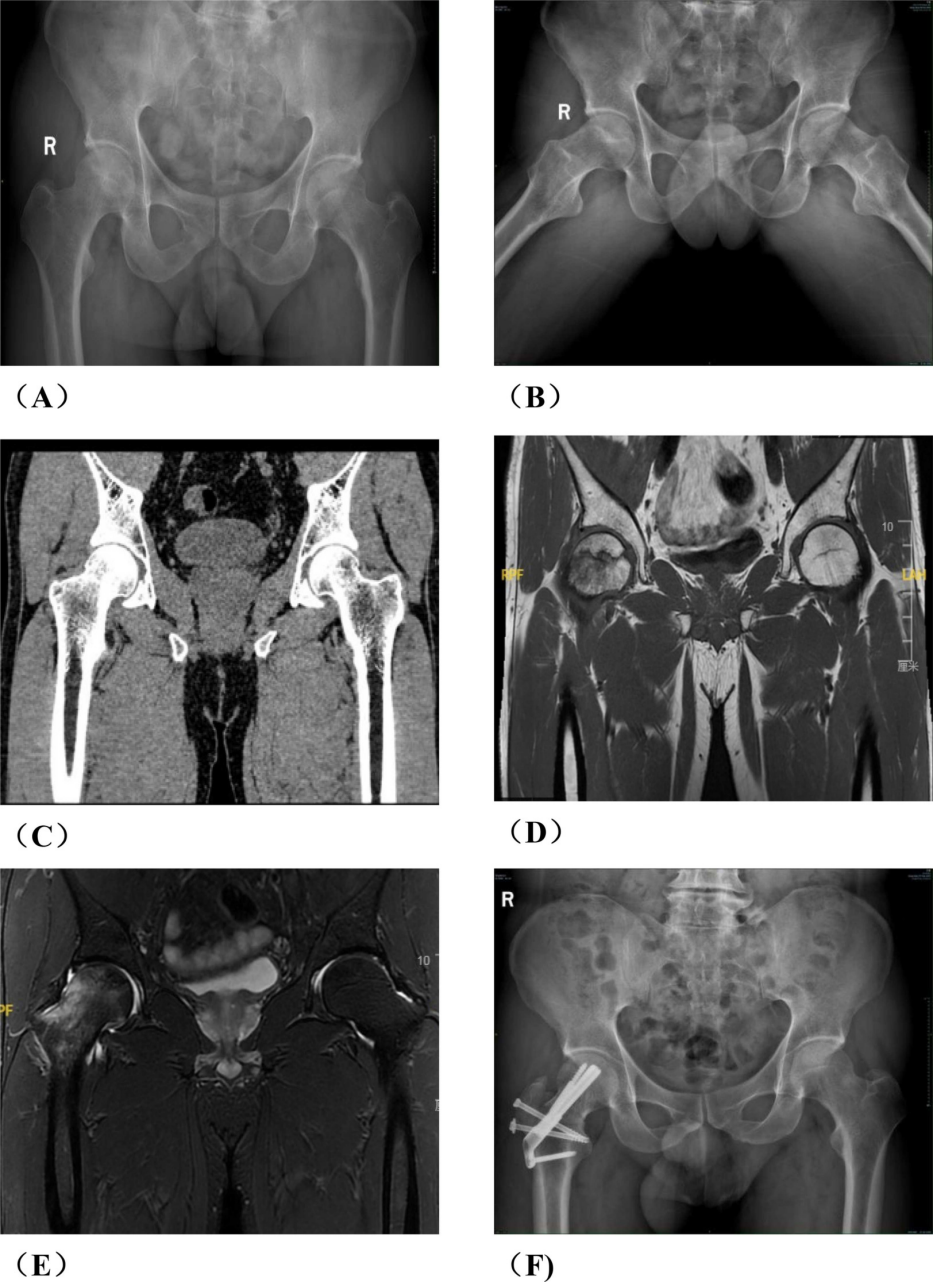
Analysis of group A patients with failed hip preservation. **(A)** Preoperative anteroposterior view; **(B)** Preoperative frog view; **(C)** Preoperative CT scan; **(E,F)** Preoperative MRI; **(G)** x-ray on the 3rd day postoperatively; **(H)** The patient returned to outpatient department for re-examination in May 2023, and radiography indicated the cannulated screws in greater trochanter were loose; **(I)** He was re-examined 3 months in August 2023 after secondary internal fixation. Radiography showed the greater trochanter was fixed; **(J)** He was re-examined 7 months after secondary internal fixation. Radiography showed the greater trochanter healed. **(K)** Postoperative TIC curve after the secondary internal fixation surgery. There was abnormal hyperperfusion in repaired area.

Group A: Liu X, male, right osteonecrosis of femoral head. The patient underwent rotational osteotomy at the base of the femoral neck under surgical hip dislocation in our hospital on March 4, 2023. One month after surgery, the patient felt a foreign body protruding from the right hip joint, accompanied with pain. So he came to our outpatient orthopedics department for a follow-up examination on May, 2023. X-ray showed that the screws in greater trochanter were loose. The patient underwent “right femoral steel needle internal fixation, loosening screw removal and local application of PRP” on May 16, 2023. After the surgery, we give the patient plaster external fixation for two months, subcutaneous injection of teriparatide, and oral administration of calcium carbonate and calcitriol. We conducted a 7-month postoperative follow-up in December 2023, and radiography showed that the loose part of the internal fixation of the greater trochanter healed. More details have been shown in Figure 9.

**Figure:**
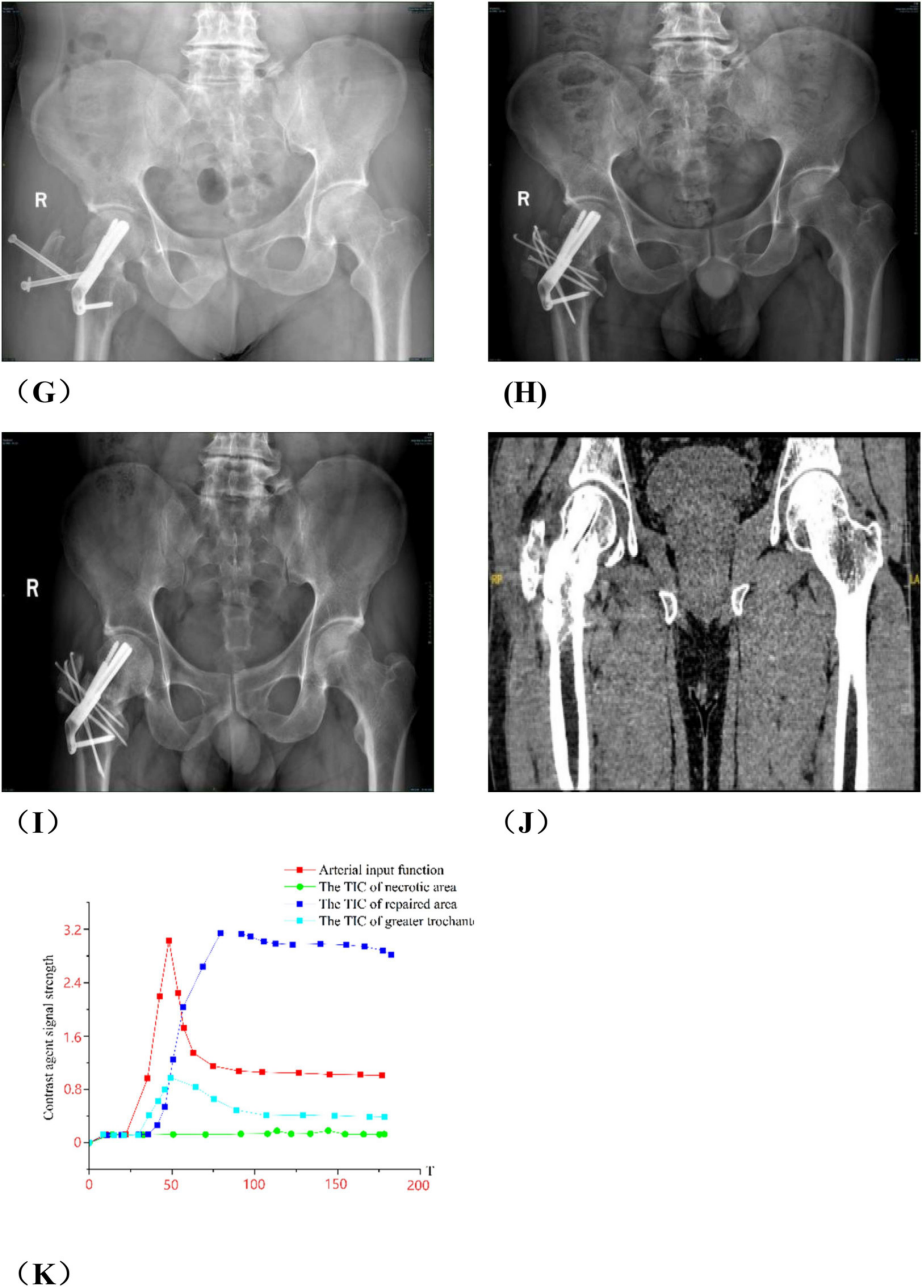


**Figure 10:**
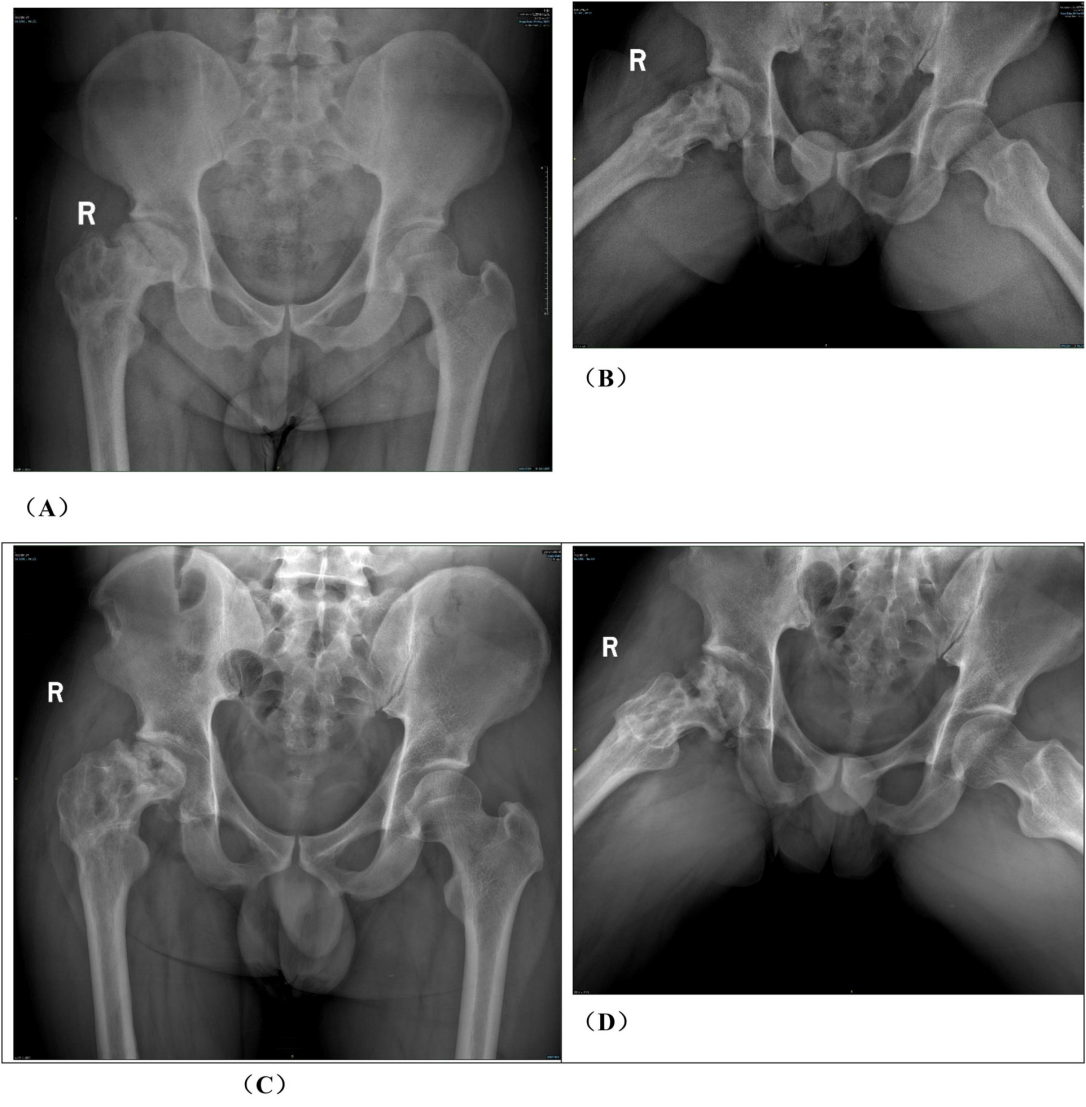
Analysis of typical patient who failed in hip preservation treatment in group B. **(A,B)** Preoperative anteroposterior and frog view: the anterolateral region was seriously involved and the weight-bearing region collapsed. **(C,D)** Postoperative anteroposterior and frog view: the iliac bone flap was not enough to support the necrotic region, and the necrosis progressed and the collapse became more serious one year after the operation. **(E)** Ktrans pseudocolor image of DCE-MRI one year after operation, showing increased perfusion in the necrotic area and abnormal hyperperfusion in the repair area. **(F)**TIC curve one year after the operation. There was abnormal hyperperfusion in repaired area. **(G–I)** The femoral head was harvested via total hip arthroplasty. We found the lateral column collapsed and the magnesium screw degraded and loose, which could be pulled out easily.

Group B: Wang XX, male, idiopathic osteonecrosis of the right femoral head. She was treated with vascularized iliac bone flap transfer. 12 months after the surgery, he felt discontented, and underwent right total hip arthoplasty. More details have been shown in Figure 10.

**Figure:**
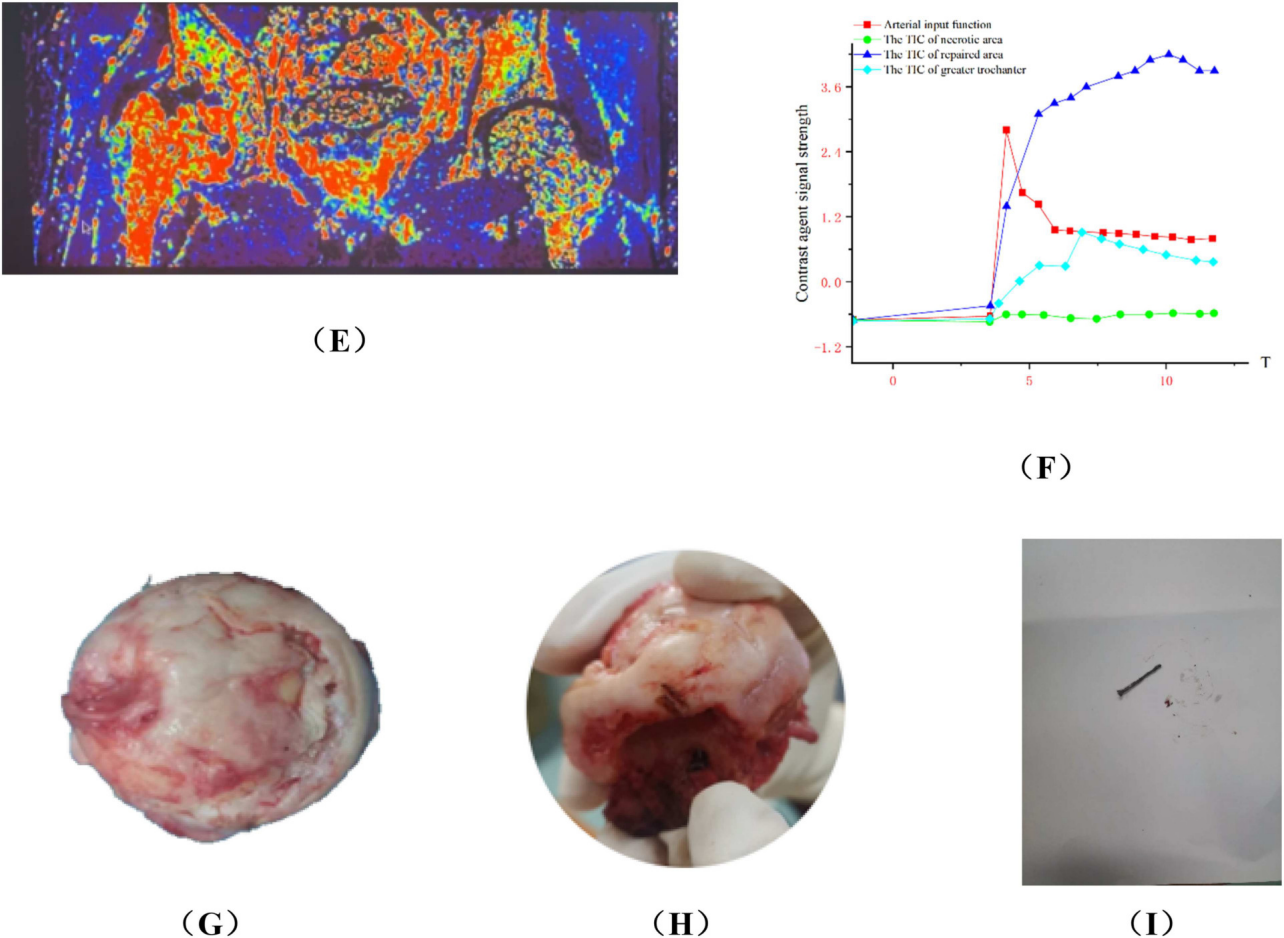


## 4 Discussion

### 4.1 Rotational osteotomy through surgical approach and vascular pedicled iliac bone flap transfer

Several critical considerations must be addressed in performing rotational osteotomy at the base of the femoral neck under surgical dislocation. Firstly, the intraoperative selection of the rotational angle is paramount, as both excessive and insufficient rotation can lead to inadequate reconstruction of the lateral column area. Traditionally, the collapsed area was identified and rotated out of the weight-bearing area subjectively during operation, so this subjective approach may lack precision. Utilizing virtual reality technology for preoperative planning can enhance the precision of the rotational angle. Notably, the surgical dislocation approach allows full exposure of the femoral head and neck (Figures 2A–E), enabling direct visualization of the anterolateral necrotic zone and accurate determination of the rotation boundary. This visualization avoids residual necrotic tissue in the weight-bearing area due to insufficient rotation or damage to the lateral column due to excessive rotation, which is crucial for ARCO ⅢB patients with severe collapse (collapse >2 mm). Secondly, given the significant surgical trauma involved, it is imperative to have a comprehensive preoperative blood preparation plan. During the procedure, meticulous care must be taken to carefully dissect blood vessels and the soft tissue flap of the external rotator muscle group, ensuring full exposure of the femoral neck base while preserving critical blood supply to the femoral head, such as the medial circumflex femoral artery. This blood supply preservation (confirmed by DCE-MRI in Figure 7 showing restored perfusion in necrotic areas) ensures that the rotated healthy bone tissue maintains viable blood flow, laying a foundation for long-term bone healing. Finally, poor alignment of the osteotomy plane can impede healing. We recommend inserting two Kirschner wires perpendicular to the femoral neck at the osteotomy line, to facilitate consistent fixation during swing-saw osteotomy.

Several critical issues must be addressed in iliac bone flap transfer with the ascending branch of the lateral femoral circumflex artery. Firstly, the bone flap must be transferred carefully due to the limited length of the iliac bone flap, and any compression or overstretching of the vascular pedicle during the operation can compromise the blood supply to graft, thereby affecting the biological reconstruction of the femoral head. Additionally, the femoral head cannot be fully exposed within the surgical field, making it difficult to completely remove necrotic area and sclerotic area. Secondly, attention must be paid to the strength of the bone graft and the positioning of the bone flap. Proper strength and correct positioning are crucial for the biomechanics of the femoral head. Lieberthal et al. (10) determined that the optimal position for the vascularized iliac bone flap is 20 degrees internally within the femoral head and anterior to the coronal plane through finite element analysis. However, the limited surgical field increases the risk of suboptimal positioning, weakening the mechanical support for the collapsed femoral head—explaining why its effect in ARCO ⅢB patients is inferior to rotational osteotomy.

### 4.2 The relationship between the effect of two kinds of operation and ARCO stage

The collapse of the femoral head is less than 2 mm in ONFH patients with ARCO IIIA stage. The internal mechanics of the femoral head still have a certain degree of stability, but self-repair is insufficient and stable repair is difficult to achieve) (1, 11). Therefore the Vascularized iliac bone flap transfer is applicable to ARCO IIIA stage. We can alleviate lateral stress and provide sufficient mechanical support to maintain the spherical shape of the femoral head via removing the necrotic bone and using an iliac bone flap supplied by the lateral circumflex femoral artery, and positioning it in the anterolateral region of the femoral head. The ascending branch of the lateral circumflex femoral artery, which has rich in blood supply, can supply fresh blood to the graft, to promote the reconstruction and induction of osteoblast formation, thereby improving the biological environment in the femoral head. This process is beneficial for restoring healthy subchondral bone and reconstructing the mechanical conduction path of the weight-bearing area (10).

The gap between the graft and the host bone was fully filled with autogenous cancellous bone and blood of bone marrow, which has excellent osteoinductive activity. This promotes better healing between the implanted cancellous bone and the femoral head. The near-normal distribution of stress within the femoral head contributes to bone remodeling and the formation of dense bone trabeculae, providing a relatively stable mechanical environment for the self-repair of the necrotic zone (6, 10). Additionally, the operation time is short, the wound is minimally invasive and the bone graft heals easily. This reduces the time required for weight-bearing and accelerates functional recovery. Therefore, we believe that vascularized iliac bone flap transfer is a superior option for patients with stage ARCO IIIA (characterized by a small area of necrosis and mild collapse).

The femoral head collapse exceeds 2 mm in patients with ARCO IIIB stage, indicating a mechanical unstable stage (1, 11). Performing a vascularized iliac bone flap transfer in such cases needs extensive debridement of necrotic bone, which significantly compromises the mechanical stability of the already collapsed femoral head. The challenges of bone resorption and the integration of subchondral and implanted bone further complicate the maintenance of biomechanical stability. Therefore, we recommend that vascularized iliac bone flap transfer be carefully considered for patients with ARCO IIIB. Conversely, rotational osteotomy of the basal portion of the femoral neck is well-suited for patients with significant but moderate necrosis collapse at ARCO IIIB stage (4). This procedure leverages the remaining healthy bone mass in the femoral head, by rotating it into the weight-bearing area to reconstruct the femoral head's mechanical conduction pathway and restore hip function. The surgical dislocation approach allows for clear exposure of the hip joint's anatomical structures, enabling complete release of the soft tissue flap on the femoral neck while preserving the blood supply to the femoral head from the medial circumflex femoral artery. Under direct visualization, the surgeon can rotate the femoral head anteriorly or posteriorly without compromising its blood supply (4, 5). Moreover, rotational osteotomy of the basal portion of the femoral neck under surgical dislocation maintains the normal anatomy of the greater and lesser trochanters. Should hip preservation fail, the complexity of performing total hip arthroplasty is reduced compared to conventional intertrochanteric rotational osteotomy (4, 12). Therefore, we believe that rotational osteotomy of the basal part of the femoral neck under surgical dislocation is a more appropriate procedure for patients with ARCO IIIB.

There are in-depth reasons for differences in efficacy between two surgical techniques. Firstly, we analyse intraoperative exposure range. The incision for the iliac bone flap transfer surgery is an SP incision. It is performed in the supine position and is made from the front of the patient. The rotational osteotomy is carried out in the lateral position, from the outside of the patient, and during the operation, the femoral head needs to be anteriorly dislocated. The lengths of the incisions for both surgeries are 10–15 cm. The iliac bone flap transfer surgery does not require dislocating the femoral head and is more minimally invasive, while the rotational osteotomy needs osteotomies in femoral neck and greater trochanter, so it is more thoroghly. Secondly, there are differences in bone flap implantation technique.the iliac bone flap is relatively small and can be firmly fixed with a single cannulated screw, so this operation is relatively easy and safe. The femoral head and femoral neck removed in the rotational osteotomy are larger and require three long cannulated screws for strong fixation, so it is relatively difficult and dangerous. Thirdly, effects and mechanisms of vascular restoration are different. The iliac bone flap transfer surgery can increase the blood supply to the femoral head via transfer of ascending branch of lateral circumflex femoral artery, while the rotational osteotomy cannot increase the blood supply to the femoral head.

### 4.3 The relationship between the effect of the two operation methods and the classification of CJFH (the location of the necrotic area)

The classification used by the Chinese-Japanese Friendship Hospital (CJFH) categorizes osteonecrosis of the femoral head based on the three-column structure developed by Professor Li Zirong (13). In our study, we observed that the CJFH-L1 lateral column was preserved, making it suitable for vascularized iliac bone flap transfer. This is primarily because the lateral column of the femoral head remains largely intact, allowing for the removal of necrotic bone with minimal risk of damaging the normal subchondral bone in the weight-bearing area. Furthermore, our findings indicated that the four patients in Group B, who underwent total hip arthroplasty after failing hip preservation attempts, were preoperatively classified as CJFH-L2 patients. This suggests that vascularized iliac bone flap transfer is less effective for those with CJFH-L2 type. The necrotic lesion is located in the lateral column and part of the central column in this type, while the medial column is typically preserved (14). We believe that the CJFH-L2 type of osteonecrosis of the femoral head is more amenable in rotational osteotomy at the base of the femoral neck under surgical dislocation, which facilitates direct reconstruction of the lateral column.

In conclusion, we conclude that the location of the necrotic area is more important than the area of the necrotic area and has a greater impact on prognosis. Therefore, the CJFH classification has a strong guiding significance for hip preservation. For different CJFFH classification of ONFH, two surgical methods can complement each other.

### 4.4 Postoperative follow-up

The osteotomy of 3 hips did not heal well 6 months after operation and healed at the last follow-up in Group A. The reasons may be as follows: (1) poor alignment and alignment after osteotomy, (2) injury of femoral head blood supply, (3) poor compliance of some patients after operation and early weight bearing. And our follow-up found that patients who underwent rotating osteotomy experienced poor healing, negative effects, and increased negative emotions. Because the patient is bedridden for a long time, the affected limb can not go to the ground to bear the weight, easy to cause muscle atrophy, hip stiffness, psychological anxiety and other problems.

Fat liquefaction occurred in 2 patients in Group B, and wound healed after debridement. We consider several reasons are followed. (1) Iliac bone flap transfer with ascending branch of lateral femoral circumflex artery is via S-P approach. If we harvest lots of cortical bone and cancellous bone near the anterior superior iliac spine, it is easy to form a subcutaneous cavity, and fluid is easy to accumulate in the cavity. This affects the healing of the incision. (2) The fenestration site is close to the skin and subcutaneous tissue, and the skin is thin. (3) There are more sweat glands near the groin. (4) The underpants are close to the incision and easily move back and forth, affect the healing of incisions near the groin. In addition, one patient felt numbness of the limb skin. We considered it is related to iatrogenic lateral femoral cutaneous nerve injury. It is suggested that the distal end of the S-P incision is close to the lateral femoral cutaneous nerve, and the lateral femoral cutaneous nerve needs to be carefully separated and protected when tensor fasciae latae and sartorius muscle space are separated during the operation.

The latest follow-up revealed that while some patients still experienced pain or discomfort, their symptoms had improved compared to preoperative levels. Some patients demonstrated what we describe as “survival with collapse” (15). Radiography showed femoral head collapsed. However, the necrotic area was well-repaired, with relatively optimal articular cartilage and joint space preserved. The femoral head and acetabulum remained well-matched and the joint stable. Previous studies have suggested that fibrous tissue proliferation within the necrotic bone trabeculae after rotational osteotomy leads to the formation of new bone structures, covering the previously necrotic trabeculae, accompanied by vascular ingrowth and a phenomenon known as ‘creeping substitution’ (16). We also observed that the cartilage and subchondral bone reintegrated together after the necrotic area was rotated to non-weight-bearing area in some patients from Group A. The Harris scores of Group B were significantly higher than those of Group A at 3 and 6 months post-operation, as well as at the last follow-up. We believe this improvement may be attributed to the vascularized iliac bone graft's active participation in the biological repair within the femoral head. Additionally, our pathological examination of the femoral heads from Group B patients who required arthroplasty indicated poor repair between the graft and the host. This suggests issues with the resorption of graft and insufficient integration with the host bone postoperatively.

### 4.5 Significance and prospects

We believe that this study offers valuable insights for future hip-preserving treatments. We recommend rotational osteotomy of the basal portion of the femoral neck under surgical dislocation for patients with CJFH-L2 and ARCO IIIB ONFH. The iliac bone flap transfer with the ascending branch of the lateral femoral circumflex artery is advantageous due to its short operation time and straightforward procedure, making it suitable for ONFH patients with CJFH-L1 type and ARCO IIIA stage.

We observed a 36% excellent rate for rotational osteotomy of the basal portion of the femoral neck under surgical dislocation in patients with ARCO Stage III necrosis of the femoral head at the last follow-up. The excellent and good rate for iliac bone flap transfer with the ascending branch of the lateral femoral circumflex artery was 44%. The long-term follow-up results for both procedures were generally moderate, requiring careful consideration when choosing surgical treatment.

Reviewing the literature (17), we found that platelet-rich plasma (PRP) therapy promotes healing at the osteotomy site and accelerates patient rehabilitation, indicating its potential for further application in later stages. Additionally, literature (18) reports that tissue engineering techniques are effective in treating ONFH. It is suggested that implanting suitable seed cells, scaffolds, and cytokines during hip-preserving surgery can exert unique biological effects, such as promoting angiogenesis, enhancing osteogenesis, and inhibiting osteoclast activity. We believe this research holds promising prospects.

### 4.6 Limitations

This study has some limitations. The sample size was relatively small, so the results of the research findings may only be applicable to patients similar to those in this study. We only conducted the research at the Jiangsu Provincial Hospital of Chinese Medicine, therefore the small sample size would amplify the impact of this bias on the results. To sum up, when extending the research conclusions to a wider range of patients, caution is needed. Moreover, the follow-up period for some patients is short. Overall, larger sample size studies with more centers, longer follow-up are needed.

## Data Availability

The original contributions presented in the study are included in the article/Supplementary Material, further inquiries can be directed to the corresponding author.
